# Mortality in Moderate to Severe Traumatic Brain Injury in Elderly Polytrauma Patients at a European Level 1 Trauma Centre—A Retrospective Cohort Study

**DOI:** 10.3390/jcm14113843

**Published:** 2025-05-29

**Authors:** Arastoo Nia, Johannes Leitgeb, Harald Kurt Widhalm, Domenik Popp, Lukas Schmoelz, Kevin Döring, David Wall, Silke Aldrian

**Affiliations:** 1 Department of Traumatology and Orthopedics, Division of Trauma Surgery, Medical University of Vienna, 1090 Vienna, Austriasilke.aldrian@meduniwien.ac.at (S.A.); 2 Department Medicine, Danube Private University, 3500 Krems, Austria; 3 Department of Traumatology and Orthopedics, Division of Orthopedic Surgery, Medical University of Vienna, 1090 Vienna, Austria; 4 Department of Anaesthesia, Intensive Care Medicine and Pain Medicine, Clinical Division of General Anaesthesia and Intensive Care Medicine, Medical University of Vienna, 1090 Vienna, Austria; david.wall@meduniwien.ac.at

**Keywords:** traumatic brain injury, polytrauma, elderly

## Abstract

**Introduction:** Traumatic brain injury (TBI) remains a significant challenge in older polytrauma patients, with age being a major determinant of outcomes. While mortality predictors have been studied in general polytrauma populations, less is known about specific risk factors in older adults with TBI. **Methods:** This retrospective study analysed data from 304 polytrauma patients over 18 years of age treated at a Level 1 trauma centre between 2013 and 2023. Patients were divided into three age categories: 18–64 years (*n* = 189), 65–84 years (*n* = 92), and ≥85 years (*n* = 23). The analysis included demographics, injury patterns, clinical indicators, surgical treatments, and in-hospital mortality to identify key mortality predictors. **Results:** The mean age was 54.5 years (SD 22.2); 72% of patients were male. In-hospital mortality was 36.3% overall, increasing to 60.8% in patients aged ≥85. TBI severity was moderate in 25% and severe in 75% of cases. Older patients were less frequently admitted to the ICU and more often managed conservatively. ICU admission was significantly lower in patients aged 65–84 (24.5%) and ≥85 (19.4%) compared to the 18–64 group (70.0%). Multivariate analysis identified age, male sex, and severe TBI as significant predictors of 30-day mortality. **Conclusions:** TBI management in older polytrauma patients requires distinct approaches due to higher mortality and poorer outcomes. Age is a critical risk factor, highlighting the need for tailored triage systems and ICU strategies to improve care and prognosis in this vulnerable population.

## 1. Background

Elderly patients with traumatic brain injury (TBI) are at high risk of experiencing disproportionate rates of mortality, longer hospital stays, and significant long-term disabilities [1]. Additionally, this group frequently has comorbidities, such as heart disease, diabetes, and dementia, and often requires treatment with antithrombotic medications. These pre-existing conditions and associated treatments complicate the management of TBIs in this population [2]. Advanced age at the time of injury is linked to poorer functional outcomes following TBI, irrespective of the severity of the injury. Mortality rates from TBI also rise significantly with age, increasing from 71% in patients aged 65–70 to 87% in those over 80 years old [3]. Recent studies show a significant rise in polytrauma from low-height falls as patient age increases, particularly among older adults, reflecting the frailty of this specific patient group despite the low-energy nature of the trauma [4].

The timely detection of severe intracranial injuries requiring specific interventions is crucial for improving outcomes. Research shows that early surgical intervention combined with intensive rehabilitation can lead to better recovery in older patients with a TBI [5]. A key challenge in managing older polytrauma patients is their tendency to initially appear clinically stable, only to deteriorate rapidly during treatment due to reduced physiological reserves. Triage systems were primarily developed for younger trauma patients, particularly those in haemorrhagic shock, and may not adequately address the needs of older patients. Due to these challenges, older patients with TBIs have often been neglected in research, as many studies on TBIs have excluded them from their analysis [6]. 

The aim of this study was, therefore, to analyse data over a 10-year period to (a) describe the epidemiology of older adults (>65 years) presenting with a TBI compared with a younger cohort (<65 years) and (b) examine variables that may be predictive of short-term outcome.

## 2. Methods

Data for this study were sourced from the Vienna Trauma Register over a 10-year span, covering the period 1 January 2013–1 December 2023. Patients included in the study were aged ≥18 years and listed in the register with a confirmed TBI. Older patients were defined as polytraumatised individuals aged >65 years. Severe TBI was characterised by an Abbreviated Injury Scale (AIS) score of ≥3 in the head region combined with a Glasgow Coma Scale (GCS) score of ≤12. Patients with mild TBI and without any evidence of head or brain injury were excluded from the study. Polytrauma was defined as a minimal Injury Severity Score (ISS) of 16.

The Vienna Trauma Register captures detailed patient data, including age, gender, GCS score, ISS, incident location, type of transport (HEMS—helicopter emergency medical service, GEMS—ground emergency medical service), brain imaging findings, treatment, various laboratory results, discharge status, and survival outcomes. The mechanism of injury (road traffic accidents, low falls [from standing] or high falls and other types of injury) was specified. Neurosurgical procedures included extra or subdural haematoma, lobectomy, and/or decompressive craniectomy. GCS measurements are typically taken upon arrival at the emergency department unless the patient was intubated, in which case the GCS score recorded at the scene of the incident was used. The Glasgow Outcome Scale (GOS) score was retrospectively assessed using patient records and neurological evaluations at 90 days. The length of stay in the intensive care unit (ICU) and three- and six-month mortality rate were recorded. The protocol was approved by the medical ethical review committee of the Medical University of Vienna, Austria (protocol number 1961/2021).

The data sets were imported into SPSS Statistics (Thomas Reuters, IBM, Version 25), and the continuous variables were graphically tested for their distribution using histograms and normal distribution curves. The distributions and location measures of the above parameters are presented in tabular form for the three age groups (18–64 years, 65–84 years and ≥85 years). The chi-square test was used for categorical variables as part of the final statistics. The Mann–Whitney U test was used for non-normally distributed metric variables. To counteract alpha error accumulation, the Bonferroni correction was then performed. The resulting *p*-values are given together with the effect size r. Here, r values < 0.3 were interpreted as small effects, between 0.3 and 0.5 as medium effects, and values > 0.5 as strong effects. The variables with a *p* value < 0.20 were included in a multivariate model, and a backwards stepwise procedure was used. The odds ratios (ORs) and 95% confidence intervals (95% Cis) were calculated. The Hosmer–Lemeshow test was used to evaluate the goodness-of-fit of the model. Numerical variables were rounded to one decimal place, except for the effect size r (2 decimal places) and the *p*-values (3 decimal places). The significance level was assumed to be *p* = 0.05. 

## 3. Results

### 3.1. Age

This study included a total of 304 polytraumatised patients. The mean age was 54.5 years (SD 22.2), and the group consisted of 219 males (72%) and 85 females (25%). The three age groups consisted of the following numbers of patients: 18–64 years (*n* = 189, 62.2%), 65–84 years (*n* = 92, 30.3%), and ≥85 years (*n* = 23, 7.5%) (Table 1).

### 3.2. Type of Injury and Severity

Falls accounted for 161 (52.9%) of all patients. There was a significant difference in injury mechanism for men and women (*p* < 0.001). Men had more high-level falls (>3 m) and road accidents. There was also a significant difference in injury mechanism between age groups (*p* < 0.001). The incidence of low-level falls increased with age. The ISS was higher for patients aged 65-84 years (32.7, *p* = 0.004, r = 0.33) and over 85, respectively (32.6). Overall, it was observed that the order of the body regions most frequently affected by severe injuries (AIS ≥ 3) was identical in both groups.

### 3.3. Anticoagulation

A significant disparity was observed in the proportion of patients receiving anticoagulants in general at the time of their accident: 66 (57.4%) of all patients aged >65 years versus 10 (5.3%) for the younger cohort (*p* > 0.001). The highest mortality rate occurred among patients undergoing anticoagulant combination therapy (*n* = 43, 56.3%, *p* = 0.9) at the time of the accident, although not significantly. 

### 3.4. Treatment and Management

Mean length of hospital stay for patients aged 65–84 years was 34 days (±49.0) and for ICU 16.2 (±15.0) days. Patient aged >85 years had the longest hospital stay, with a mean of 35 days, and shortest ICU stay (mean 14.4 days). The mean length of hospital and ICU stay for patients aged <65 years was 25.8 days (±47.4) and 14.5 (±13.0). The differences between the age groups differed regarding length of hospital stay and ICU, but not significantly (*p* = 0.3 and 0.6, respectively) (Table 1 and Table 2).

Of the 151 patients with subdural haematoma, 84 had a neurosurgical intervention. Patients who received neurosurgery were younger (*p* = 0.04), with a mean age of 75.6 (SD 7.0) years versus 80.9 (SD 8.2) years, and were more likely to be male (*p* = 0.02). Outcomes for those having neurosurgical intervention were significantly better (18.7% mortality) than for those without surgery (30.5% mortality, *p* < 0.001). 

### 3.5. Neurological Outcome

In patients aged 65–100 years, the GOS was 2.9 ± 1.3, with a median of 3.0. Among patients < 64 years, the mean GOS was higher, at 4.1 ± 1.6 (*p* < 0.001, r = 0.45) (Table 3).

### 3.6. Mortality

**Figure 1:**
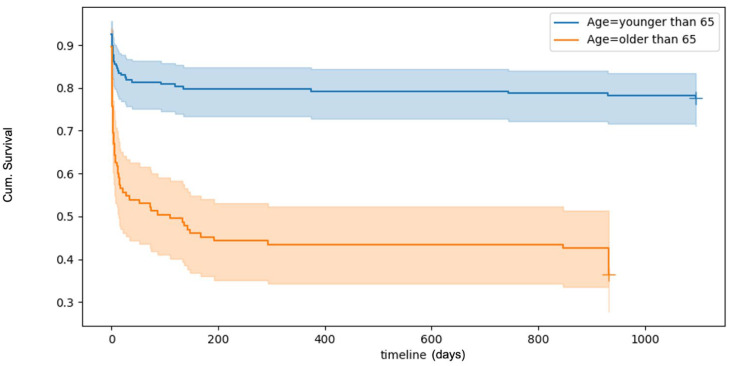
Kaplan–Meyer plot of overall survival after TBI. The plot shows a poorer survival rate with increased patient age.

Survival analyses, visualised using Kaplan–Meier curves (Figure 1), revealed a 30-day overall mortality rate of 32.9%. Mortality rates increased with age. Logistic multivariate regression analyses identified several factors significantly associated with an increased risk of death within 30 days post-trauma. These included increasing age, male sex, and severe TBI. ICU and baseline ASA were not found to influence 30-day survival outcomes (Table 4).

## 4. Discussion

Our study provides insights into the epidemiology and outcomes of polytraumatized traumatic brain injuries of old patients over the past 10 years. We were able to demonstrate a correlation between age, TBI severity, sex, and higher mortality rates, and specifically in patients between 65 and 84 years of age. This finding aligns with previous studies indicating that elderly trauma patients face elevated mortality risks, particularly when presenting with severe injuries. Furthermore, older patients might benefit from ICU admission and surgical intervention. These observations underscore the critical importance of tailored management triage and treatment strategies for severely injured elderly patients to improve outcomes in this vulnerable population.

Upon arrival at the shock room, older patients’ mean GCS score was significantly higher than that of younger patients. This difference was even more pronounced when comparing median GCS values, with older patients having a mean of 14.2 versus 9.7 in the younger group. Research suggests that older adults with the same anatomical severity of TBI often exhibit a higher GCS score compared with younger patients [7]. This difference may stem partly from age-related brain atrophy, which allows larger haematomas and more significant oedema to develop before clinical symptoms become evident [8]. Consequently, this can result in under-triaging of older patients and may also account for the reduced effectiveness of triage tools in this population, therefore resulting in worse outcome [9]. Frailty screening may be used as a possibility not only for risk stratification but also to improve patient outcomes [10]. Frailty screening can serve not only as a tool for risk assessment but also as a means to enhance outcomes for patients > 65 years.

More than half (57.4%) of polytrauma patients > 65 years were on oral anticoagulants or antiplatelet agents at the time of their injury. However, their mortality rates did not significantly exceed those of older patients who were not on such medication. While some studies have suggested that anticoagulants may elevate mortality in older trauma patients by exacerbating risks, such as intracranial haemorrhages and general bleeding, others found no clear link between anticoagulation and worse outcomes. However, for traumatic brain injuries, stronger evidence connects anticoagulant or antiplatelet use to poorer prognoses [11].

Several studies have linked the use of anticoagulants or antiplatelet drugs to poorer outcomes or higher mortality in trauma patients. Karni et al. highlighted a lethal risk associated with the combination of anticoagulation therapy, age > 65, and head trauma due to intracranial haemorrhages [12]. Ivascu et al. showed the effects of an aggressive anticoagulation reversal protocol in patients previously on coumarins, which led to a 75% reduction in mortality linked to traumatic intracerebral haemorrhage in older patients [13].

### 4.1. Surgical Intervention

Research shows a growing trend towards less aggressive treatment in older patients with a TBI [14]. High mortality and morbidity in this group may contribute to a self-fulfilling prophecy, influencing early surgical decisions. Research on neurosurgical outcomes in older patients remains inconclusive, with some studies reporting improved survival and function after surgery, while others suggest better outcomes with conservative management [15,16,17]. Survival after neurosurgical intervention in this study was significantly higher compared with conservative treatment. Nevertheless, in this study, younger patients with severe head injuries were more likely to undergo surgical intervention than their older counterparts (21.7% versus 5.9%). This disparity can, in part, be attributed to ongoing debate about the risks and benefits of surgical interventions in older patients with a TBI [14]. Time and age could be a possible explanation for the low numbers in the group > 65 years, as older patients often experience delays in medical intervention due to underestimating the danger and therefore causing a wait-and-see attitude in healthcare staff. ASA scores were not significantly different between the age groups; however, concomitant disease in older patients might have influenced the decisions. Although early intervention can improve survival rates, as seen in studies where prompt surgery reduced mortality from 90% to 30%, such benefits are less pronounced in the elderly due to their fragile physiological state [18]. In this study, older individuals had higher survival rates with surgery versus conservative treatment, but due to the small sample size of the studied groups, no recommendations for the timing of a neurosurgical intervention can be derived and, therefore, this needs to be further investigated. 

### 4.2. Intensive Care Unit Management

The ICU admission of elderly patients remains a topic of debate. However, advancements in neurosurgical procedures and modern neurointensive care have improved outcomes for older patients with a TBI [19]. Merzo et al. showed that up to 55% of patients aged 70–79 and up to 30% of patients aged ≥80 years with a TBI achieved a favourable neurological outcome [20].

The analysis of ICU stays revealed comparable mean durations for older and younger polytrauma patients, with stays of 14.5 and 16.2 nights, respectively. Patients > 85 years stayed 14.4 days in the ICU. However, durations for all age groups were notably longer than those reported in other studies, suggesting that institutional practices, patient populations or injury severity may have affected the results. For example, Giannoudis et al. reported significantly shorter ICU stays, with a median of eight days for both age groups, highlighting potential differences in care protocols or regional healthcare systems [21]. A particularly important finding was the significantly lower proportion of older patients admitted to the ICU compared with their younger counterparts. While younger patients were frequently treated in intensive care following stabilisation in the shock room, many patients > 65 years were transferred directly to normal wards without ICU monitoring.

### 4.3. Overall Mortality

In this study, older polytrauma patients exhibit mortality rates that are significantly higher than 30 days compared with younger patients. These findings align with Giannoudis et al., who reported mortality rates of 42% in older patients compared with 20% in younger groups, with mortality rising to nearly 50% in patients > 75 years [21]. DeMaria et al. also highlighted increased mortality in older patients (≥80 years) compared with patients aged 65–79 years, while Richmond et al. noted that mortality risk escalates by approximately 5% with each additional year of age [22,23]. Age-related physiological changes also lower the threshold for traumatic stress, making older patients particularly susceptible to poor outcomes, even in cases of mild TBI [24,25]. 

### 4.4. Neurological Results

Neurological and functional outcomes, assessed using the GOS at discharge, were significantly worse in patients > 65 years. These findings are consistent with studies reporting poorer outcomes for older patients with a TBI [26]. King et al. demonstrated that patients with a GOS of three months post-trauma improved to GOS 4–5 within a year following intensive rehabilitation. These findings emphasise the potential for recovery and the importance of comprehensive long-term care strategies for older polytrauma patients [27]. However, in other areas of patient care for adults > 65 years, such as hip fractures, prompt acute management has been found to significantly reduce hospital stays and mortality rates, even for patients aged ≥60 with notable comorbidities (19). However, this study found that older patients with a TBI might experience reduced surgical interventions, have fewer admissions to the ICU and face extended hospital stays along with higher mortality rates. As recent findings suggest that early and aggressive treatment of TBIs in older adults can be as effective as it is for younger patients (4), greater emphasis and further research on this population is warranted.

### 4.5. Limitations

This study does have some limitations, due to its retrospective design. It focused exclusively on patients with a TBI. In addition, relevant data on underlying medications and comorbidities were not available. Knowledge of comorbidities is important, since patients with chronic comorbidities and TBI especially have different clinical needs than patients with only TBI. Saviter et al. showed a 2.07 times greater risk among older individuals for nosocomial infections, which is even more important since this is an independent predictor of poor global outcomes in severe TBI up to 5 years postinjury [28,29].

Due to the small sample size of 23 patients aged over 85 years, the results for this group are questionable, even though other authors have reported similar findings. The study’s retrospective design and small sample size further limit the generalisability of its findings. GOS outcome data were collected at three months, but the possibility of further improvement beyond this period cannot be ruled out. In fact, it has been suggested that functional status is best assessed at six months or later. Furthermore, frailty was not assessed in the study patients; frailty is now acknowledged as a key factor influencing outcomes, particularly in older ICU patients [30].

## 5. Conclusions

The range of severe and costly outcomes associated with TBI in older adults further underscores the critical importance of addressing this issue. While age remains an important consideration in therapy and intensive care, it should not be the sole determining factor. Greater emphasis should be placed on a patient’s functional capacity, anticipated quality of life, and individual preferences. These aspects should play a central role in the decision-making process, particularly in light of evolving patient demographics

## Figures and Tables

**Table 1:** Demography and clinical and surgical variables.

	18–64 yrs	65–84 yrs	≥85 yrs	*p*-Value
Count (*n*)	189	92	23	
Male sex (*n*, %)	141 (74.6)	50 (54.3)	14 (60.9)	0.005
Pre-injury ASA-score mean	1.2	2.1	2.2	<0.001
Pre-injury antithrombotics (*n*, %)				
Marcoumar	4 (2.1)	11 (12.0)	6 (26.1)	0.79
Platelet inhibitor	3 (1.6)	35 (38.0)	2 (8.7)	<0.001
Platelet inhibitor + anticoagulation	3 (1.6)	10 (11.0)	2 (8.7)	<0.001
Injury mechanism (*n*, %)				
Fall > 3 m	55 (29.1)	6 (6.5)	0	0.008
Fall < 3 m	13 (6.9)	69 (75.0)	18 (78.3)	<0.001
Road traffic accident	68 (36.0)	13 (14.1)	5 (21.7)	0.009
Other	53 (28.0)	4 (4.3)	0	
GCS (mean)	5.7	6.2	6.7	0.4
ISS (mean)	26.4	32.7	32.6	0.004
Injured body region AIS ≥ 3				
Head and neck	113 (59.8)	88 (95.7)	21 (91.3)	<0.001
Thorax	85 (45.0)	9 (9.8)	2 (2.2)	0.006
Extremities	57 (30.2)	6 (6.5)	1 (1.1)	0.4
Abdomen	38 (20.1)	2 (2.2)	0	0.3
Face	11 (5.8)	1 (1.1)	0	0.2
Skin	4 (2.1)	0	0	0.1
Type of transport (*n*, %)				
GEMS without doctor	98 (51.9)	60 (65.2)	18 (78.3)	0.8
GEMS with doctor	40 (21.2)	6 (6.5)	3 (13.0)	0.6
HEMS	51 (27.0)	26 (28.3)	2 (8.7)	0.5
Mean length of stay in hospital (days) (SD)	25.8 ± 47.4	34.1 ± 49.0	35.0 ± 77.1	0.3
Intubation on arrival (*n*, %)	89 (47.1)	23 (25.0)	5 (21.7)	0.08

**Table 2:** ICU data and mortality.

	18–64 yrs	65–84 yrs	≥85 yrs	*p*-Value
ICU Transfer	132 (70.0)	23 (24.5)	5 (19.4)	<0.001
Mean length of ICU stay in days (SD)	14.5 (±13.0)	16.2 (±15.0)	14.4 (±7.0)	0.6
Mortality (*n*, %)				
30 days mortality	35 (18.5)	53 (57.6)	12 (52.2)	<0.001
6 month mortality	38 (20.1)	64 (69.7)	13 (56.2)	<0.001

**Table 3:** Neurological data and outcomes.

	18–64 yrs	65–84 yrs	≥85 yrs	*p*-Value
Pathoanatomy (*n*, %)				
Skull fracture	76 (40.2)	42 (45.7)	7 (28.4)	0.1
Large subdural haemorrhage (>1 cm)	35 (18.5)	9 (9.8)	3 (13.0)	0.04
Small subdural haemorrhage (<1 cm)	80 (42.3)	20 (21.7)	4 (17.4)	0.009
Surgical Management (*n*, %)				
Osteoclastic trepanation	34 (18.0)	6 (6.5)	0	0.09
Osteoplastic trepanation	17 (9.0)	3 (3.2)	0	0.07
Borehole trepanation	15 (7.9)	5 (5.4)	4 (17.4)	0.009
GOS (*n*, %)				
Good neurological outcome (GOS 4–5)	120 (63.5)	31 (33.7)	6 (26.1)	<0.001
Poor neurological outcome (GOS 2–3)	31 (16.4)	19 (20.7)	5 (21.7)	0.08

**Table 4:** Logistic multivariate regression of parameters potentially associated with risk of death within 30 days of trauma.

	OR	95% CI	*p*-Value
Age	3.31	1.86–5.91	<0.001
Male sex	2.86	1.34–4.56	<0.001
Preinjury ASA	1.09	1.02–1.10	0.4
ICU stay	1.07	1.02–1.10	0.9
TBI severity	2.65	1.58–4.44	<0.001

## Data Availability

The data presented in this study are available on request from the corresponding author.
